# Prediction of Exercise Tolerance in the Severe and Extreme Intensity Domains by a Critical Power Model

**DOI:** 10.5114/jhk/170101

**Published:** 2023-09-05

**Authors:** Thiago Pereira Ventura, Fernando Klitzke Borszcz, Diego Antunes, Fabrizio Caputo, Tiago Turnes

**Affiliations:** 1 Physical Effort Laboratory, Sports Center, Federal University of Santa Catarina, Florianopolis, Brazil.; 2 Human Performance Research Group, Center for Health Sciences and Sport, Santa Catarina State University, Florianopolis, Brazil.

**Keywords:** maximal oxygen uptake, cycling, high-intensity exercise, power-duration relationship

## Abstract

This study aimed to assess the predictive capability of different critical power (CP) models on cycling exercise tolerance in the severe- and extreme-intensity domains. Nineteen cyclists (age: 23.0 ± 2.7 y) performed several time-to-exhaustion tests (Tlim) to determine CP, finite work above CP (W'), and the highest constant work rate at which maximal oxygen consumption was attained (I_HIGH_). Hyperbolic power-time, linear power-inverse of time, and work-time models with three predictive trials were used to determine CP and W'. Modeling with two predictive trials of the CP work-time model was also used to determine CP and W'. Actual exercise tolerance of I_HIGH_ and intensity 5% above I_HIGH_ (I_HIGH+5%_) were compared to those predicted by all CP models. Actual I_HIGH_ (155 ± 30 s) and I_HIGH+5%_ (120 ± 26 s) performances were not different from those predicted by all models with three predictive trials. Modeling with two predictive trials overestimated Tlim at I_HIGH+5%_ (129 ± 33 s; p = 0.04). Bland-Altman plots of I_HIGH+5%_ presented significant heteroscedasticity by all CP predictions, but not for I_HIGH_. Exercise tolerance in the severe and extreme domains can be predicted by CP derived from three predictive trials. However, this ability is impaired within the extreme domain.

## Introduction

Critical power (CP) delimitates the boundary between heavy- and severe-intensity domains and represents the highest intensity of exercise in which some physiological and metabolic responses achieve a steady state (Black et al., 2017). During exercise in the severe domain, oxygen consumption (VO_2_) kinetics presents a slow component, increasing the O_2_ cost of exercise and leading VO_2_ to the maximum value (VO_2max_) before exhaustion (Jones et al., 2011). Conversely, in the extreme intensity domain (i.e., supra-severe exercises), although VO_2_ response seems to be faster, exercise exhaustion precedes the attainment of VO_2max_ (Burnley and Jones, 2007). Therefore, the maximal intensity at which VO_2max_ can be achieved before exhaustion (I_HIGH_) indicates the boundary between severe- and extreme-intensity domains. However, the mechanisms that define exercise tolerance between these domains remain uncertain.

CP and the finite work capacity above CP (*W'*) can be derived from mathematical models based on the power-duration relationship within the severe intensity domain (Hill, 1993; Poole et al., 2016). While the hyperbolic model (CP_hyp_) provides CP from the asymptote of the hyperbola, and the curvature constant denotes *W'*, these variables may also be obtained by a linear relationship between work × time (CP_linear_) or power × inverse of time (CP_1/time_) (Hill, 1993; Monod and Scherrer, 1965; Moritani et al., 1981). These models allow for the prediction of time-to-exhaustion (Tlim) at intensities above CP, which theoretically would correspond to the moment of *W'* depletion (Chidnok et al., 2013).

Exercise tolerance during the severe intensity domain is compromised by the magnitude of the slow component of VO_2_ (VO_2SC_) kinetics, which would be explained by the inefficiency of muscle fibers to maintain exercise intensity and increasing O_2_ cost, leading VO_2_ to VO_2max_ (Burnley and Jones, 2007; Jones et al., 2011). Murgatroyd et al. (2011) found a positive correlation between *W'* and the magnitude of VO_2SC_ (r^2^ = 0.76). Therefore, in the severe intensity domain, *W'* depletion coincides with the moment of attainment of VO_2max_, leading to exercise interruption (Murgatroyd et al., 2011). However, in the extreme intensity domain, VO_2SC_ is not pronounced, and VO_2max_ is not reached, because the exhaustion precedes VO_2max_ attainment (Burnley and Jones, 2007). Thus, *W'* is not completely depleted in the extreme domain, and the exercise tolerance prediction could be impaired (Alexander et al., 2019). Nevertheless, the prediction of exercise tolerance in intensities at which occurs the transition of severe to extreme intensity domain was not completely investigated. Therefore, it is not known whether there is an exact point at which the prediction of exercise tolerance begins to fail.

In the extreme intensity domain, Alexander et al. (2019) reported a higher slope of the linear relationship between power and the inverse of time (1/time), culminating in lower *W'* in the extreme compared to the severe domain in knee extension exercise. In the study by Alexander et al. (2019), exercise tolerance in the extreme domain was overestimated by variables of the power-time relationship of the severe domain (CP and *W*’), except for the work rate that was considered the ‘transition point’ between the severe and the extreme intensity domain. However, those findings are restricted to knee extension exercise. In addition, the “transition point” between the severe and extreme domains was estimated as bouts with time to exhaustion shorter than 2 min (Alexander et al., 2019). Therefore, a valid measure to discriminate between the severe and extreme intensity domains (i.e., VO_2max_ attainment) could be insightful to assess the accuracy of exercise tolerance prediction in the transition point between these domains and support understanding the physiological determinants of whole-body exercise tolerance around these domains.

The ability of CP and *W'* to predict exercise tolerance at intensities within the severe domain has been verified (Chidnok et al., 2013; Jones et al., 2008; Morgan et al., 2019; Nimmerichter et al., 2020). However, there is a gap in the literature about the prediction of exercise tolerance at intensities close to the “transition point” between severe and extreme intensity domains (Alexander et al., 2019; Caputo and Denadai, 2008; Charkhi Sahl Abad et al., 2021). Thus, the main aim of this study was to assess the prediction of cycling exercise tolerance in the boundary between severe and extreme intensity domains by different CP models. The main hypothesis was that CP models would accurately predict exercise tolerance in the severe intensity domain, but this predictability could be reduced in the extreme intensity domain.

## Methods

### Participants

Nineteen male subjects (mean ± standard deviation [SD]; age: 23.0 ± 2.7 years; body mass: 77.8 ± 6.2 kg; body height: 175.3 ± 5.3 cm; and peak oxygen uptake [VO_2peak_]: 49.4 ± 5.6 mL∙kg^−1^∙min^−1^) classified as recreationally trained cyclists (De Pauw et al., 2013) participated in the study and gave their written informed consent. The study was approved by the Institutional Ethics Committee for Research on Human Subjects from the State University of Santa Catarina and was performed according to the Declaration of Helsinki. Participants were instructed to continue normal daily activities during the study period.

### Procedures

**Figure 1:**
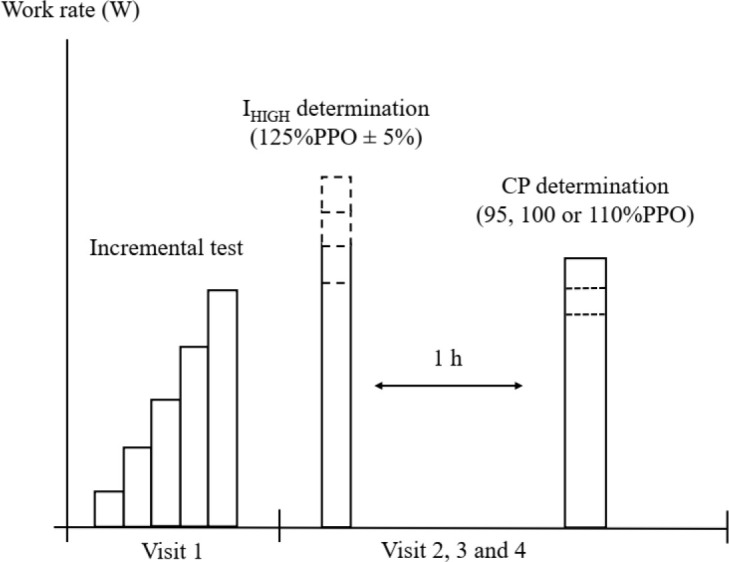
Schematic illustration of the experimental design. I_HIGH_: the high constant work rate at which VO_2max_ is attained; PPO: peak power output; CP: critical power

All participants visited the laboratory at least four times on different days for testing (Figure 1). On the first day, participants performed an incremental test to determine the peak power output (PPO) and VO_2peak_ of the incremental test. During their second, third, and fourth visits, in random order and on separate days, participants performed constant Tlim tests (95%, 100%, and 110% of PPO) to determine CP and *W'*. Before CP predictive trials, separated by a 1-h passive rest interval, participants completed two to three Tlim tests on separate days to determine the maximal intensity at which VO_2max_ can be achieved (I_HIGH_) as well as the work rate 5% above it (I_HIGH+5%_) as previously published (Turnes et al., 2016). The 60-min of passive rest proved to be sufficient to allow full recovery of *W'* and minimize any potential priming effect (Muniz-Pumares et al., 2019). All exercise tests were preceded by a standardized 10-min warm-up and a 5-min passive rest as described elsewhere (Turnes et al., 2016). All tests were separated by ≥24 h within 14 days and were performed at the same time of day to minimize the effects of diurnal biological variation on results. Participants were also asked to refrain from consuming caffeine and arrive at the laboratory for at least 2 h after the last meal before each trial.

### Materials

All exercise tests were conducted using an electronically braked cycle ergometer (Lode Excalibur Sport, Groningen, The Netherlands). During all tests, the pulmonary gas exchange was measured breath-by-breath using an automated open-circuit gas analysis system (Quark PFT, COSMED, Rome, Italy). Before each test, the gas analyzer was calibrated using ambient air and gases containing 16% oxygen and 5% carbon dioxide. The turbine flow meter used for the determination of minute ventilation was calibrated with a 3-L calibration syringe (COSMED, Rome, Italy). The heart rate was also monitored throughout the tests (Polar, Kempele, Finland).

### Incremental Test

The initial power output for the incremental test was set at 0.5 W∙kg^−1^ for 3 min and then increased by 0.5 W∙kg^−1^ every 3 min until voluntary exhaustion (Caputo and Denadai, 2008; Moseley and Jeukendrup, 2001). Participants were instructed to maintain their preferred cadence between 70 and 90 rotations per minute (rpm) for as long as possible. PPO was defined as the power output attained at exhaustion if the test was terminated at the end of a 3-min stage. If the test was terminated before the last stage had finished, PPO was calculated as the power of the previous stage plus the power increment multiplied by the duration of exercise in the final stage divided by 180 s (Kuipers et al., 1985). VO_2peak_ of the incremental test was defined as the highest average VO_2_ over a 15-s period (Robergs et al., 2010).

### Critical Power Protocol

For the determination of CP and *W'*, three constant work rate tests in random order were performed. Power outputs for these trials were equivalent to 95, 100, and 110% of PPO, estimated to produce a Tlim between 3 and 9 min (Caputo and Denadai, 2008). VO_2peak_ during the constant work rate trials was defined for each test as the highest average VO_2_ over a 15-s period (Robergs et al., 2010). Time-to-exhaustion was recorded to the nearest second. Using three distinct two-parameter models, four combinations of CP and *W'* were estimated as follows:

- CP_linear_: from the linear time-work model using three predictive trials (95, 100, and 110% of PPO) (Hill, 1993).
- CP_linear(95,110)_: from the linear time-work model using two predictive trials (95 and 110% of PPO) (Hill, 1993).
- CP_1/time_: from the linear power × inverse of the time model using three predictive trials (95, 100, and 100% of PPO) (Hill, 1993).
- CP_hyp_: from the hyperbolic 2-parameter model with three predictive trials (95, 100, and 110% of PPO) (Hill, 1993).

The prediction of the CP_1/time(95,110)_ model was omitted from further analysis because it provided identical values to CP_linear(95,110)_.

Exercise tolerance of I_HIGH_ and I_HIGH+5%_ was predicted by all CP and *W'* models employing the CP_hyp_ equation.

### The Boundary between Severe and Extreme Exercise Intensities

All participants performed two to three Tlim tests to determine I_HIGH_ (severe domain) and I_HIGH+5%_ (extreme domain), beginning at 125% of PPO (Turnes et al., 2016). When VO_2max_ could be reached or maintained during the first Tlim test, further subsequent constant Tlim tests at a 5% higher work rate were performed on separate days until VO_2max_ could not be reached. On the other hand, when VO_2max_ could not be reached or maintained during the first Tlim test, further Tlim tests were conducted at a 5% lower work rate. I_HIGH_ was defined for each participant as the highest power output at which the highest 15-s VO_2_ average (determined from rolling averages of 5-s samples) was equal to or higher than VO_2max_ (averaging the highest VO_2peak_ values from the incremental and CP predictive trials), minus one intraindividual standard deviation (SD) (4.0% ± 1.4%); i.e., SD derived for each participant’s VO_2peak_ from incremental and CP predictive trials tests (Turnes et al., 2016). I_HIGH_ was individually determined and considered the last intensity of the severe domain, while I_HIGH+5%_ was the first intensity of the extreme domain.

### Statistical Analysis

The descriptive statistics are presented as means ± SD and statistical variables as mean point estimates with confidence intervals of 95% (95% CI). Agreements between actual and predicted Tlim at I_HIGH_ and I_HIGH+5%_ tests were assessed by Bland-Altman analyses with bias and 95% limits of agreement (LoA) (Bland and Altman, 1999). Linear regression was performed to verify homoscedasticity (constant dispersion of differences across the range of averages) or heteroscedasticity (increase or decrease in dispersion as the averages increase) between the actual and predicted measures on the Bland-Altman plots (Ludbrook, 2010). Additionally, ANOVA for repeated measures was used to compare CP and *W'* among the models and actual and predicted Tlim at I_HIGH_ and I_HIGH+5%_. When an ANOVA significant main effect was observed, post hoc tests with corrections of Tukey’s were applied between CP and *W'* estimates, while comparisons between actual and predicted Tlim at I_HIGH_ and I_HIGH+5%_ were made using the Dunnet test. We conducted a sensitivity analysis in G*Power (version 3.1.9.7, Düsseldorf, Germany) to determine the smallest effect that one could have detected with high probability given n = 19, *p* < 0.05, and statistical power = 95%. In the current study, we obtained ANOVA F-values of 2.8 for CP and *W'*, 2.5 for Tlim, and 3.3 for VO_2max_. Statistical analyses were performed with the software GraphPad Prism 8.1.2 (GraphPad Software, La Jolla, CA, USA). The statistical significance level was established at *p* < 0.05.

## Results

The PPO of the incremental test was 274 ± 35 W and the Tlim of CP predictive trials was 424 ± 48, 310 ± 37, and 223 ± 23 s for 95%, 100%, and 110% PPO, respectively. VO_2max_ (i.e., the average VO_2peak_ of incremental and CP predictive trials: 3.75 ± 0.41 L∙min^−1^) was not significantly different from VO_2peak_ of I_HIGH_ (3.72 ± 0.46 L∙min^−1^), but significantly higher than VO_2peak_ of I_HIGH+5%_ (3.50 ± 0.41 L∙min^−1^; F_(1.7, 31)_ = 38.0; *p* < 0.0001). For I_HIGH+5%_ determination, no participants attained VO_2max_ during the test.

There were no significant differences among the models for CP (F_(1.3, 24)_ = 3.0; *p* = 0.088) or W' (F_(1.3, 23)_ = 3.8; *p* = 0.052, Table 1).

**Table 1:** Critical power and *W'* estimates for the different models.

	CP_linear(95,110)_	CP_linear_	CP_1/time_	CP_hyp_
CP (W)	211 ± 39	213 ± 38	212 ± 38	213 ± 39
SEE (%)	-	4.5 ± 4.3	5.2 ± 5.1	4.7 ± 4.6
*W'* (kJ)	20.3 ± 5.7	19.4 ± 5.4	19.9 ± 5.4	19.4 ± 6.1
SEE (%)	-	15.0 ± 12.2	14.4 ± 13.3	16.7 ± 13.4
R^2^	-	0.996 ± 0.006	0.966 ± 0.047	0.973 ± 0.032

Data are in mean ± SD.

CP: critical power; SEE: standard error of estimate; W': finite work capacity above critical power

The mean power output of I_HIGH_ and I_HIGH+5%_ was 344 ± 52 and 371 ± 53 W, respectively. Comparisons between actual and predicted Tlim at I_HIGH_ and I_HIGH+5%_ by CP and W' models showed no significant ANOVA main effects for I_HIGH_ (F_(1.4, 26)_ = 2.1; *p* = 0.157), but a significant main effect was found for I_HIGH+5%_ (F_(1.7, 31)_ = 4.6; *p* = 0.023). Pairwise comparisons demonstrated a significant difference between actual and predicted Tlim at I_HIGH+5%_ by the CP_linear(95,110)_ model (*p* = 0.030), with no significant differences for the CP_linear_ (*p* = 0.268), CP_1/time_ (*p* = 0.072), and CP_hyp_ (*p* = 0.512) models (Table 2).

**Table 2:** Actual and predicted time-to-exhaustion at I_HIGH_ and I_HIGH+5%_ by different CP and *W'* models.

Variables		Actual Tlim	Predicted by CP and *W'* models			
			CP_linear(95,110)_^a^	CP_linear_	CP_1/time_	CP_hyp_
**I_HIGH_ Tlim**						
	Time (s)	155 ± 30	154 ± 33	150 ± 34	152 ± 33	149 ± 36
	Bias ± SD_diff_ [LoA] (s)	- -	−0.5 ± 13.3 [−26.6 to 25.6]	−4.8 ± 15.0 [−34.2 to 24.6]	−2.8 ± 13.5 [−29.3 to 23.7]	−5.8 ± 18.8 [−42.7 to 31.1]
	Bias ± SD_diff_ [LoA] (%)	- -	−0.7 ± 8.1 [−16.6 to 15.2]	−3.8 ± 9.2 [−21.9 to 14.3]	−2.3 ± 8.3 [−18.5 to 13.9]	−4.8 ± 11.7 [−27.7 to 18.1]
**I_HIGH+5%_ Tlim**						
	Time (s)	120 ± 26	129 ± 33*	125 ± 33	127 ± 32	124 ± 35
	Bias ± SD_diff_ [LoA] (s)	- -	9.2 ± 13.7 [−17.7 to 36.1]	5.3 ± 13.0 [−20.2 to 30.8]	7.2 ± 12.6 [−17.5 to 31.9]	4.6 ± 15.3 [−25.4 to 34.6]
	Bias ± SD_diff_ [LoA] (%)	- -	6.6 ± 9.3 [−11.7 to 24.9]	3.3 ± 9.2 [−14.7 to 21.3]	5.1 ± 8.6 [−11.7 to 21.9]	2.4 ± 11.1 [−19.3 to 24.1]

Actual and predicted performances are shown in means ± SD.

LoA: limits of agreement of 95% (i.e., SD_diff_ × 1.96), SD_diff_: standard deviation of the differences

between actual and predicted performance, Tlim: time-to-exhaustion, 95% CI: confidence intervals of 95%.

a provides the same results as the CP_1/time(95,110)_ model

* Significantly different from actual Tlim (p = 0.036)

**Figure 2:**
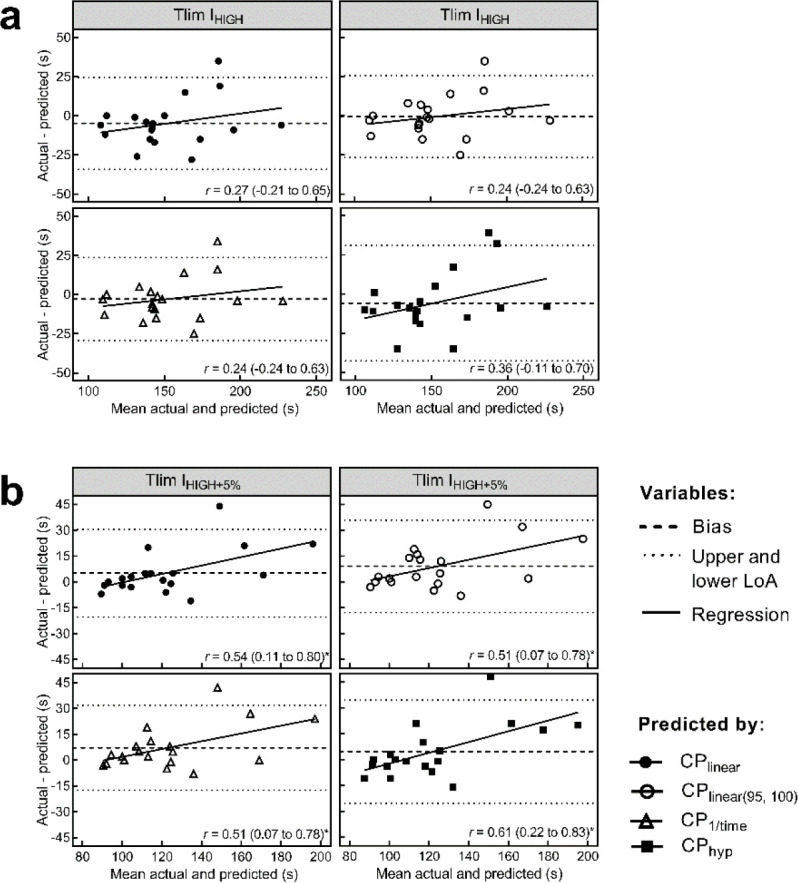
Bland-Altman plot with linear regression for actual and predicted time-to-exhaustion of I_HIGH_ (a) and I_HIGH+5%_ (b) by all models of power-time relationship variables. The dashed line indicates the mean difference error. Dotted lines indicate lower and upper limits of agreement. Solid lines are the regressions between the bias and average values accompanied by correlation coefficients (r) with 95% CI to detect homoscedasticity or heteroscedasticity. * p < 0.05.

Bland-Altman plots for I_HIGH_ and I_HIGH+5%_ are presented in Figure 2. The bias ± 95% LoA in raw and percent units are presented in Table 2. Moderate heteroscedasticity was observed only for I_HIGH+5%_ in all models (Figure 2).

## Discussion

This study aimed to evaluate the prediction of cycling exercise tolerance in the boundary between severe and extreme intensity domains by different power-duration models. According to the main hypothesis, all models predicted Tlim within the severe domain (i.e., I_HIGH_). Conversely, in partial disagreement with the hypothesis, exercise tolerance within the extreme intensity domain (i.e., I_HIGH+5%_) was not statistically different from that predicted by CP and *W'* estimates derived from three predictive trials. However, the heteroscedasticity observed at I_HIGH+5%_ indicated that the prediction of exercise tolerance was impaired when increasing exercise intensity towards the extreme domain. Furthermore, since the model with only two predictive trials affected the estimate of exercise tolerance during the extreme intensity domain, caution is required when utilizing this model, especially for short exercise duration.

Critical power with three predictive trials was able to predict exercise tolerance at the beginning of the extreme domain (i.e., I_HIGH+5%_), which partially refutes the main hypothesis that distinct physiological mechanisms could explain exhaustion in the severe and extreme intensity domains. This could theoretically be explained by the fact that the transitions between intensity domains are not exact points (i.e., thresholds), but a phase of modifications (Pethick et al., 2020). Alexander et al. (2019), during knee extension exercise, suggested that additional factors to those observed in the severe domain could explain exhaustion in the extreme domain. Those authors observed that exercises in the extreme intensity domain lasting ~55, ~37, and ~27 s were overestimated by CP and *W'* derived by severe-domain work rates. In addition, they showed exclusive *W'* for the extreme domain (1.7 ± 0.4 kJ), which was less than *W'* of the severe domain (5.9 ± 1.5 kJ). However, they did not report differences between predicted and actual Tlim at the work rate that would demarcate the transition from the severe to the extreme intensity domain (i.e., 60% 1 RM, average Tlim 85 s).

Likely, exercise tolerance prediction in work rates towards the boundary of severe and extreme intensity domains is still sensitive to variables estimated in the severe domain. Different from CP that demarcates a threshold between heavy and severe intensity domains and presents distinct responses in metabolic and neuromuscular variables in work rates slightly below and above CP, I_HIGH_ does not seem to indicate a threshold that presents substantial differences in these responses (Iannetta et al., 2022). Nevertheless, the increased heteroscedasticity observed herein suggests this predictability can be impacted at work rates in the upper zones of extreme domains, as observed by Alexander et al. (2019).

The findings demonstrated relative precision of CP and *W'* models to predict exercise tolerance in the severe intensity domain, with a bias between −0.7% and −4.8% for I_HIGH_, which appears to be better than the 5.7% to 9.4% mean bias between models to predict 5-km running time-trial performance (Nimmerichter et al., 2017). However, these values were similar to the mean bias of 2.9% and 1.3% of the best individual fit of CP to predict 16.1-km (Morgan et al., 2018) and 20-min (Nimmerichter et al., 2020) cycling time trials, respectively. Interestingly, the CP_linear(95,110)_ model underestimated the actual exercise tolerance for I_HIGH_ by only −0.7%, with a lower bias than models with three predictive trials (Morgan et al., 2018; Nimmerichter et al., 2017, 2020). However, the prediction of exercise tolerance presented high individual variability (i.e., LoA), ranging from ±15.9% to ±22.9%, which is substantially superior to the LoA of ±4.6% to ±6.7% reported by Nimmerichter et al. (2020).

In contrast, in the extreme intensity domain, the concordance analysis showed that, on average, the models overestimated exercise tolerance at I_HIGH+5%,_ with an average bias between 2.4% and 6.6%. Furthermore, LoA values for I_HIGH+5%_ ranged from ±16.8% to ±21.7%. Therefore, despite a significant difference between actual and predicted exercise tolerance at I_HIGH+5%_ only for the CP_linear(95,110)_ model, the findings indicate that CP and *W'* models tended to underestimate exercise tolerance at the upper intensity of the severe domain and overestimate Tlim at the lower intensity of the extreme domain, with high interindividual variability in both.

Some factors may contribute to the high LoA values found herein in the severe intensity domain compared to previous reports (Morgan et al., 2018; Nimmerichter et al., 2020). First, it could be the type of performance to determine CP, since the time-trial test has a lower variation in test-retest reliability (Laursen et al., 2007) and lower SEE for both CP and *W'* (Karsten et al., 2017) compared to the Tlim test, which seems to result in greater precision to estimate these variables and consequently, better accuracy of performance prediction. Second, the number of predictive trials to determine CP and *W'* should be considered. There is a tendency to decrease SEE with the use of more predictive trials, depending on duration (Matunara et al., 2018), which also leads to better precision in estimating these variables due to the best mathematical modeling fit. Third, the training status of participants should be taken into account. Studies carried out with trained cyclists (Morgan et al., 2018; Nimmerichter et al., 2020) and runners (Nimmerichter et al., 2017) have described a low variability between predicted and actual performance values. This was not found in the present study, exhibiting an effect of familiarity with maximal effort tests on performance prediction in others (Morgan et al., 2018; Nimmerichter et al., 2017, 2020). It is possible that these factors were also determinants of the high LoA values found in the present study.

The linear regression analyses in the Bland-Altman plots (i.e., homoscedasticity or heteroscedasticity verification) for I_HIGH_ and I_HIGH+5%_ (Figures 2a and 2b) provide interesting information on the factors that would be involved in the tolerance to exercise at intensities above CP. While the agreement between actual and predicted Tlim for I_HIGH_ demonstrates homoscedasticity, the moderate heteroscedasticity was found in the analysis of agreement between actual and predicted Tlim for I_HIGH+5%_. The homoscedasticity found in I_HIGH_ indicates that, even in participants in which CP models overestimated the actual Tlim, there was no tendency of the mean differences to increase as the time of the task increased. On the other hand, at the intensity 5% above (i.e., I_HIGH+5%_), which represents the first intensity of the extreme domain in this study, the linear regression indicated that there was a significant influence of the task duration on the prediction error by CP models, in which participants who had higher Tlim also had the most overestimated prediction by models, which may denote an influence of the predominance of anaerobic metabolism in the prediction of exercise tolerance. As observed by Alexander et al. (2019) in single-joint exercise, there was a tendency for the CP and *W'* models to overestimate Tlim exercise in the extreme domain of cycling. This corroborates the hypothesis that different physiological mechanisms would be involved in exercise exhaustion and, therefore, reinforces the existence of a supra-severe intensity domain. However, there seems to be a zone of intensities at which full depletion of *W'* can still be used to predict exercise tolerance in the extreme domain, questioning which factors would be involved in determining this variable.

The present study is not free of limitations. The duration of predictive trials to determine CP and *W'* was relatively short (424 ± 48, 310 ± 37, and 223 ± 23 s for 95%, 100%, and 110% of PPO, respectively), which could affect the estimation of these variables. However, these times are included in the range recommended between 3 and 12 min by Muniz-Pumares et al. (2019) and utilized by others (Caputo and Denadai, 2008; Turnes et al., 2016). Furthermore, although the SEE of the power-duration relationship was acceptable for CP (~4.5%), it was relatively high for *W'* (14%), which is higher than the recommended 10% (Hill, 1993; Muniz-Pumares et al., 2019) and can influence the predictions made here. Although three predictive trials are sufficient to estimate CP and *W'* for 2-parameter models (Caputo and Denadai, 2008; Hill, 1993), the inclusion of more points in work-time modeling could reduce SEE. The small sample size is also a relevant limitation that should be recognized, especially for Bland-Altman and homoscedasticity/heteroscedasticity analyses.

## Conclusions

In conclusion, exercise tolerance at intensities near the boundary between severe and extreme intensity domains can be predicted by CP and *W'* derived from three predictive trials. However, heteroscedasticity analyses and the disagreement observed between the actual and the predicted exercise tolerance when increasing exercise intensity demonstrate that CP predictive ability is reduced at higher work rates of the extreme intensity domain.

## References

[ref1] Alexander, A. M., Didier, K. D., Hammer, S. M., Dzewaltowski, A. C., Kriss, K. N., Lovoy, G. M., & Barstow, T. J. (2019). Exercise tolerance through severe and extreme intensity domains. Physiological Reports, 7(5), e14014. 10.14814/phy2.14014 30825269 PMC6397101

[ref2] Black, M. I., Jones, A. M., Blackwell, J. R., Bailey, S. J., Wylie, L. J., McDonagh, S. T., ... & Vanhatalo, A. (2017). Muscle metabolic and neuromuscular determinants of fatigue during cycling in different exercise intensity domains. Journal of Applied Physiology, 122(3), 446–459. 10.1152/japplphysiol.00942.2016 28008101 PMC5429469

[ref3] Bland, J. M., & Altman, D. G. (1999). Measuring agreement in method comparison studies. Statistical Methods in Medical Research, 8(2), 135–160. 10.1177/096228029900800204 10501650

[ref4] Burnley, M., & Jones, A. M. (2007). Oxygen uptake kinetics as a determinant of sports performance. European Journal of Sport Science, 7(2), 63–79. 10.1080/17461390701456148

[ref5] Caputo, F., & Denadai, B. S. (2008). The highest intensity and the shortest duration permitting attainment of maximal oxygen uptake during cycling: effects of different methods and aerobic fitness level. European Journal of Applied Physiology, 103(1), 47–57. 10.1007/s00421-008-0670-5 18196264

[ref6] Charkhi Sahl Abad, A., Ghram, A., Soori, R., Akbarnejad, A., Azizi Ghuchan, F., & Zare, M.M. et al. (2021). Purslane supplementation lowers oxidative stress, inflammatory and muscle damage biomarkers after high-intensity intermittent exercise in female runners. Balt J Health Phys Activ, 13, 17-27. 10.29359/BJHPA.13.1.03. PMC13587602 42761812

[ref7] Chidnok, W., Dimenna, F. J., Bailey, S. J., Wilkerson, D. P., Vanhatalo, A., & Jones, A. M. (2013). Effects of pacing strategy on work done above critical power during high-intensity exercise. Medicine and Science in Sports and Exercise, 45(7), 1377–1385. 10.1249/mss.0b013e3182860325 23377832

[ref8] De Pauw, K., Roelands, B., Cheung, S. S., De Geus, B., Rietjens, G., & Meeusen, R. (2013). Guidelines to classify subject groups in sport-science research. International Journal of Sports Physiology and Performance, 8(2), 111–122. 10.1123/ijspp.8.2.111 23428482

[ref9] Hill, D. W. (1993). The critical power concept. Sports Medicine, 16(4), 237–254. 10.2165/00007256-199316040-00003 8248682

[ref10] Iannetta, D., Zhang, J., Murias, J. M., & Aboodarda, S. J. (2022). Neuromuscular and perceptual mechanisms of fatigue accompanying task failure in response to moderate-, heavy-, severe-, and extreme-intensity cycling. Journal of Applied Physiology (Bethesda, Md. : 1985), 133(2), 323–334. 10.1152/japplphysiol.00764.2021 35771217

[ref11] Jones, A.M., Wilkerson, D.P., Vanhatalo, A., & Burnley, M. (2008). Influence of pacing strategy on O_2_ uptake and exercise tolerance. Scandinavian Journal of Medicine and Science in Sports, 18(5), 615–626. 10.1111/j.1600-0838.2007.00725.x 18067518

[ref12] Jones, A. M., Grassi, B., Christensen, P. M., Krustrup, P., Bangsbo, J., & Poole, D. C. (2011). Slow component of VO_2_ kinetics: mechanistic bases and practical applications. Medicine & Science in Sports & Exercise, 43(11), 2046–62. 10.1249/MSS.0b013e31821fcfc1 21552162

[ref13] Karsten, B., Hopker, J., Jobson, S. A., Baker, J., Petrigna, L., Klose, A., & Beedie, C. (2017). Comparison of inter-trial recovery times for the determination of critical power and W’ in cycling. Journal of Sports Sciences, 35(14), 1420–1425. 10.1080/02640414.2016.1215500 27531664

[ref14] Kuipers, H., Verstappen, F. T. J., Keizer, H. A., Geurten, P., & Van Kranenburg, G. (1985). Variability of aerobic performance in the laboratory and its physiologic correlates. International Journal of Sports Medicine, 6(04), 197–201. 10.1055/s-2008-1025839 4044103

[ref15] Laursen, P. B., Francis, G. T., Abbiss, C. R., Newton, M. J., & Nosaka, K. (2007). Reliability of time-to-exhaustion versus time-trial running tests in runners. Medicine and Science in Sports and Exercise, 39(8), 1374–1379. 10.1249/mss.0b013e31806010f5 17762371

[ref16] Ludbrook, J. (2010). Confidence in Altman–Bland plots: a critical review of the method of differences. Clinical and Experimental Pharmacology and Physiology, 37(2), 143–149. 10.1111/j.1440-1681.2009.05288.x 19719745

[ref17] Maturana, F. M., Fontana, F. Y., Pogliaghi, S., Passfield, L., & Murias, J. M. (2018). Critical power: how different protocols and models affect its determination. Journal of Science and Medicine in Sport, 21(7), 742–747. 10.1016/j.jsams.2017.11.015 29203319

[ref18] Monod, H., & Scherrer, J. (1965). The work capacity of a synergic muscular group. Ergonomics, 8(3), 329–338. 10.1080/00140136508930810

[ref19] Morgan, P. T., Black, M. I., Bailey, S. J., Jones, A. M., & Vanhatalo, A. (2019). Road cycle TT performance: Relationship to the power-duration model and association with FTP. Journal of Sports Sciences, 37(8), 902–910. 10.1080/02640414.2018.1535772 30387374

[ref20] Moritani, T., Nagata, A., Devries, H. A., & Muro, M. (1981). Critical power as a measure of physical work capacity and anaerobic threshold. Ergonomics, 24(5), 339–350. 10.1080/00140138108924856 7262059

[ref21] Moseley, L., & Jeukendrup, A. E. (2001). The reliability of cycling efficiency. Medicine and Science in Sports and Exercise, 33(4), 621–627. 10.1097/00005768-200104000-00017 11283439

[ref22] Muniz-Pumares, D., Karsten, B., Triska, C., & Glaister, M. (2019). Methodological approaches and related challenges associated with the determination of critical power and curvature constant. Journal of Strength & Conditioning Research, 33(2), 584–596. doi: 10.1519/JSC.0000000000002977 30531413

[ref23] Murgatroyd, S. R., Ferguson, C., Ward, S. A., Whipp, B. J., & Rossiter, H. B. (2011). Pulmonary O_2_ uptake kinetics as a determinant of high-intensity exercise tolerance in humans. Journal of applied physiology, 110(6), 1598–1606. 10.1152/japplphysiol.01092.2010 21415174

[ref24] Nimmerichter, A., Novak, N., Triska, C., Prinz, B., and Breese, B. C. (2017). Validity of treadmill-derived critical speed on predicting 5000-meter track-running performance. Journal of Strength and Conditioning Research, 31(3), 706–714. 10.1519/JSC.0000000000001529 27379951

[ref25] Nimmerichter, A., Prinz, B., Gumpenberger, M., Heider, S., & Wirth, K. (2020). Field-Derived Power–Duration Variables to Predict Cycling Time-Trial Performance. International Journal of Sports Physiology and Performance, 15(8), 1095–1102. 10.1123/ijspp.2019-0621 32040941

[ref26] Pethick, J., Winter, S. L., & Burnley, M. (2020). Physiological evidence that the critical torque is a phase transition, not a threshold. Medicine and Science in Sports and Exercise, 52(11), 2390. 10.1249/MSS.0000000000002389 32366801 PMC7556242

[ref27] Poole, D. C., Burnley, M., Vanhatalo, A., Rossiter, H. B., & Jones, A. M. (2016). Critical power: an important fatigue threshold in exercise physiology. Medicine and Science in Sports and Exercise, 48(11), 2320. 10.1249/MSS.0000000000000939 27031742 PMC5070974

[ref28] Robergs, R. A., Dwyer, D., & Astorino, T. (2010). Recommendations for improved data processing from expired gas analysis indirect calorimetry. Sports Medicine, 40(2), 95–111. 10.2165/11319670-000000000-00000 20092364

[ref29] Turnes, T., de Aguiar, R. A., de Oliveira Cruz, R. S., Pereira, K., Salvador, A. F., & Caputo, F. (2016). High-intensity interval training in the boundaries of the severe domain: Effects on sprint and endurance performance. International Journal of Sports Medicine, 37(12), 944–951. 10.1055/s-0042-109068 27551939

